# Exploring Coping Strategies and Quality of Life in Adolescents with Cancer: Pilot Study Findings

**DOI:** 10.3390/children12101312

**Published:** 2025-09-29

**Authors:** Monica Licu, Darren Haywood, Elisabeta Nita, Adrian Pogacian

**Affiliations:** 1 Department of Ethics and Academic Integrity, Faculty of Medicine, Carol Davila University of Medicine and Pharmacy, 050474 Bucharest, Romania; monica.licu@umfcd.ro; 2 Human Performance Research Centre, Insight Research Institute, School of Sport, Exercise and Rehabilitation, University of Technology Sydney, Ultimo, NSW 2007, Australia; darren.haywood@uts.edu.au; 3 Fundeni Clinical Institute, 022328 Bucharest, Romania; 4 Incka Psycho-Oncology Center, 540124 Targu-Mures, Romania; adipogacian@gmail.com

**Keywords:** adolescents, cancer, quality of life, coping, psychosocial support

## Abstract

**Highlights:**

**What are the main findings?**

- Adolescents with cancer reported a moderate-to-good perceived quality of life (mean PedsQL score ≈ 70).
- Emotion-focused and avoidance-based coping strategies (acceptance, distraction, social withdrawal) were the most frequently used.

**What is the implication of the main finding?**

- Findings provide preliminary evidence that adolescents demonstrate adaptive capacity but rely heavily on avoidance strategies.
- Psychosocial interventions may need to promote active, problem-focused coping to enhance resilience and well-being.

**Abstract:**

Objective: The objective of this exploratory pilot study was to examine the relationship between coping strategies and perceived quality of life in adolescents diagnosed with oncological diseases, with attention to the potential role of psychosocial factors in emotional adaptation. Method: The study included 20 adolescents (12 boys, 8 girls), aged 12–18 years, enrolled in the hospital school program in Bucharest, Romania, while receiving active oncological treatment. Participants completed two validated instruments: the Pediatric Quality of Life Inventory (PedsQL—Cancer Module) and the KidCOPE questionnaire. Results: The mean quality of life score was 70, indicating a moderately good level of quality of life. Emotion-focused and avoidance-based strategies (distraction, social withdrawal, and acceptance) were most frequently reported, while problem-focused coping was less common. Regression analysis showed that coping dimensions explained approximately 26% of the variance in quality of life (R^2^ = 0.26, F(3,16) = 1.83, *p* = 0.183). Although the overall model was not statistically significant, an observed negative association was found between avoidant coping and quality of life (*p* = 0.037). These results should be interpreted with caution given the small sample size and cross-sectional design. Discussion: The findings suggest that adolescents with cancer may maintain a functional level of adaptation despite medical and emotional challenges, supported by medical staff and social resources. The predominance of avoidant strategies highlights the need for further investigation of their long-term implications. Conclusions: These preliminary results generate hypotheses and underline the importance of future research on psychological and educational interventions aimed at fostering more active coping strategies and supporting resilience in adolescents with cancer.

## 1. Introduction

Quality of life is a multidimensional and highly subjective construct that reflects how individuals perceive their position in life in the context of the culture and value systems in which they live, as well as their personal goals, expectations, and concerns [1,2]. In pediatric oncology, quality of life is of particular significance, as it is shaped not only by the somatic dimensions of the disease, but also by its psychological and social repercussions on children’s or adolescents’ development [3]. Specialized literature proposes an integrative model of quality of life encompassing five essential dimensions: physical, material, emotional, social, and functional well-being [4,5]. Maintaining a functional state close to normal poses a major challenge for pediatric cancer patients. Aggressive treatments, frequent hospitalizations, and disruption of daily routines significantly impact emotional balance and the overall quality of life [6,7].

Adolescents (aged 12–18 years) navigating a complex phase of identity formation and emotional growth employ a variety of psychological coping mechanisms in response to serious illnesses, such as cancer. The coping strategies they adopt, whether adaptive or maladaptive, play a vital role in how effectively they manage stress and preserve their positive self-perception [8]. Moreover, factors such as social support, disease awareness, and family context substantially influence adolescents’ psychological adjustment to their traumatic experience of cancer [9,10].

In this context, the present pilot study aimed to investigate the relationship between coping strategies and perceived quality of life among adolescents diagnosed with cancer in Romania, and to examine the influence of psychosocial factors. Given the distinctive clinical and educational environment, including the hospital school program that provides educational continuity during active treatment, this study contributes novel data from an underrepresented population. Rather than drawing firm clinical recommendations, the study is intended as exploratory and hypothesis-generating, offering a preliminary understanding of the emotional needs of this population and informing directions for future research.

## 2. Method

### 2.1. Objectives and Hypotheses

The objective was to explore how adolescents diagnosed with cancer adapt psychologically and emotionally to their illness within the broader framework of biopsychosocial influences. The following hypotheses were formulated:

1. Adolescents with oncological conditions would predominantly use emotion-focused and avoidance-based coping strategies.
2. Coping strategies may account for variance in perceived quality of life.
3. Adaptive coping strategies were expected to show a positive association with higher levels of perceived quality of life.

### 2.2. Participants

The study sample consisted of 20 adolescents (12 males [60%] and 8 females [40%]) aged between 12 and 18 years (mean age = 15.3 years, SD = 2). All participants had oncological diagnoses and were enrolled in the hospital school program (Bucharest, Romania) while receiving active treatment in a pediatric oncology unit. The inclusion criteria were as follows: confirmed oncological diagnosis, cognitive capacity to understand and complete self-report instruments, absence of severe neurocognitive impairment, and provision of informed consent by legal guardians. Adolescents provided informed assent by voluntarily completing the anonymous survey.

### 2.3. Measures

Pediatric Quality of Life Inventory (PedsQL—Cancer Module): An internationally validated instrument comprising 27 items organized across multiple dimensions, including pain, nausea, procedural anxiety, treatment-related anxiety, cognitive difficulties, perceived physical appearance, and communication [11]. Scores were converted to a scale from 0 to 100, with higher values indicating a better perceived quality of life. In the present sample, internal consistency was excellent (Cronbach’s α = 0.94).

KidCOPE (10 items): A self-report questionnaire assessing coping strategies used by children and adolescents in stressful situations [12]. For the purpose of this study, responses were grouped into four main categories: active coping, emotion-focused coping, avoidant coping, and maladaptive coping. The participants rated the frequency and perceived effectiveness of each strategy. Internal consistency for the total scale in this sample was excellent (Cronbach’s α = 0.90).

### 2.4. Procedure

This cross-sectional survey, with a descriptive-observational pilot design, was conducted in February 2025. All participants completed the anonymous questionnaires in a supportive environment that ensured comfort, privacy, and clarity of instructions under the supervision of trained medical staff. Parents or legal guardians provided informed consent prior to participation, and adolescents assented by voluntarily completing the questionnaires. The average completion time was 20–25 min. Ethical standards concerning confidentiality, informed consent, and the right to withdraw were strictly followed. Approval was obtained from the Ethics Committee of the Fundeni Clinical Institute (No. 2876/27 January 2025).

## 3. Results

**Figure 1:**
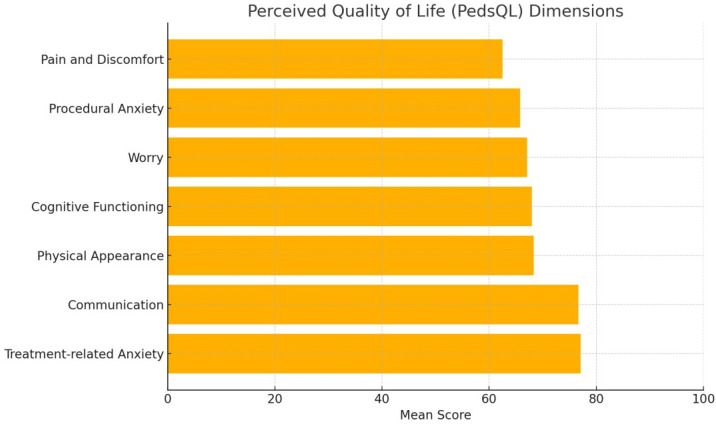
Perceived Quality of Life (PedsQL) Dimensions.

**Figure 2:**
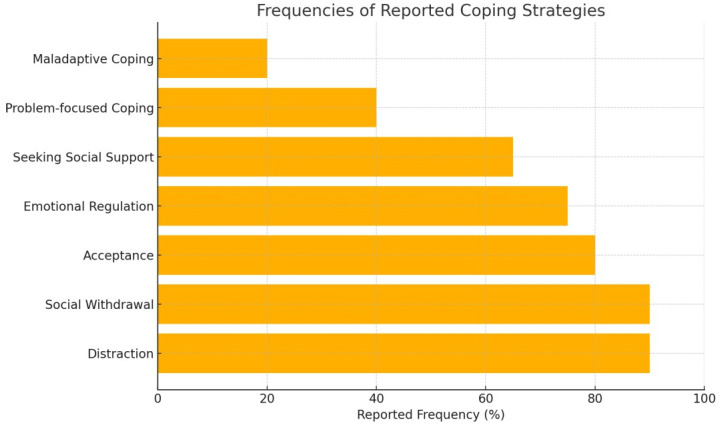
Frequencies of Reported Coping Strategies.

The overall mean score on the Pediatric Quality of Life Inventory (PedsQL) was 70, suggesting a relatively good quality of life among the adolescents in this sample. Figure 1 and Figure 2 illustrate the distribution of coping strategies, indicating that acceptance, distraction, and social withdrawal were most frequently reported. This pattern is consistent with a predominant use of emotion-focused and avoidance-based strategies, providing preliminary support for Hypothesis 1.

In the multiple regression model, the dependent variable was the total quality of life score (PedsQL), while the independent variables were the coping dimensions (adaptive, avoidant, emotional). The model accounted for approximately 26% of the variance, but this effect did not reach statistical significance (R^2^ = 0.26, F(3,16) = 1.83, *p* = 0.183); therefore, Hypothesis 2 was not supported. Within the model, avoidant coping showed a negative association with quality of life (β = −31.49, *p* = 0.037), while adaptive coping showed a positive but non-significant trend (β = 19.62, *p* = 0.081). Emotional coping was not significantly associated with quality of life (β = 10.98, *p* = 0.352) (see Table 1).

## 4. Discussion

The analysis of data on adolescent cancer patients maintaining functional balance despite the difficulties related to the disease, treatments, and the emotional impact of hospitalization is supported by international specialized literature. The moderate-to-good quality of life score (approximately 70) was somewhat unexpected but aligned with evidence that many AYAs can maintain functional balance despite the challenges of cancer. Studies have shown that adolescents and young adults (AYAs) diagnosed with cancer can maintain a satisfactory functional level owing to adapted psychosocial interventions and effective social support [13]. Interventions that promote autonomy and decision-making, provide intimacy, and facilitate social/peer interactions are more effective in improving psychosocial outcomes [14,15].

Adolescent patients with cancer use a variety of coping strategies, including cognitive avoidance and emotional regulation. These strategies reflect both age-specific characteristics and the need for psychological protection in the face of major stressors. Studies have shown that positive cognitive coping strategies are associated with more favorable attitudes toward the disease, while defensive strategies may have the opposite effect [16,17]. Social support is essential for adolescents and young adults undergoing cancer treatment. This contributes to maintaining a form of normality and developing independence, strong peer relationships, and identity exploration [18]. All sources of social support should be informed about these developmental needs in order to participate in useful and appropriate behaviors that help AYA patients cope with cancer [19]. The low frequency of problem-focused strategies indicates the possible need for educational and psychological interventions to increase the sense of control and autonomy in the face of the disease. These findings provide preliminary indications that supportive interventions may play a role in maintaining functional balance and perceived quality of life, though further research is needed to confirm these associations.

The results underline that adolescents’ coping strategies cannot be interpreted solely based on their frequency of use, but also by considering the perceived effectiveness of those strategies. In the case of positive reappraisal, the discrepancy between the effect of frequency and perceived effectiveness suggests that simply applying this strategy, without the belief that it works, may be associated with a negative impact on quality of life. Conversely, when adolescents perceive positive reappraisal as useful, it is linked to an increase in quality of life, highlighting the cognitive role of internal validation in the adaptive process [20].

Strategies focused on active problem-solving and self-soothing appear to be consistently beneficial, supporting the literature that identifies them as protective mechanisms against stress and psychosocial risk factors [21,22]. These behaviors involve both cognitive components (planning, analysis) and emotional components (self-regulation, tension reduction), making them effective in supporting well-being.

Interestingly, rumination—often considered a risk factor for mental health—showed a positive correlation with quality of life in this sample. This unexpected result should be interpreted cautiously, as it may reflect cultural or contextual differences or be an artifact of the small sample size. Recent studies also highlight that behavioral risk factors contribute to overall well-being and adaptation in oncology patients, underlining the importance of considering lifestyle factors alongside psychosocial support [23].

## 5. Clinical Implications

The results of this study highlight the importance of integrating psychosocial interventions that focus not only on emotional support but also on enhancing adolescents’ sense of control and competence. Interventions should foster problem-solving skills, improve illness-related knowledge, and promote constructive emotional regulation strategies. The inclusion of peer-support models may also facilitate emotional expression and normalization of the illness experience. Given that avoidant coping showed a negative correlation with quality of life, interventions might consider targeting the reduction in maladaptive avoidance and the strengthening of adaptive strategies. These recommendations should be regarded as tentative until confirmed in larger studies.

## 6. Limitations and Directions for Future Research

This study has several limitations. The small sample size and the cross-sectional design limit the generalizability of the findings and preclude causal inferences. Furthermore, the self-report nature of the instruments may introduce bias due to social desirability or differing levels of self-awareness. Additionally, this study did not assess socio-economic background, educational level, gender-related factors, type or stage of cancer, or broader cultural and health system influences, all of which may impact coping and quality of life. Future research should consider longitudinal designs, larger and more diverse samples, and include qualitative components to capture the nuanced emotional experiences of adolescents living with cancer. As this was a pilot study, the small sample size should be interpreted as exploratory, providing preliminary evidence rather than definitive conclusions.

## 7. Conclusions

The oncological adolescents in the sample perceived quality of life at a moderate to good level, highlighting the positive relationships with medical staff and acceptance of treatment, while physical pain and procedural anxiety remain important challenges. Coping mechanisms were predominantly oriented towards emotional avoidance and affective support. It is necessary to promote problem-solving strategies and psychosocial interventions adapted to support resilience, self-efficacy, and emotional development. As a pilot study, these findings are exploratory and should be viewed as preliminary evidence. They may offer tentative guidance for designing psychosocial programs aimed at fostering resilience, self-efficacy, and healthy emotional development in adolescent oncology patients. Broader cultural and health system factors, as well as identity-related variables, should be considered in future larger and longitudinal studies to validate and extend these results.

## Figures and Tables

**Table 1:** Multiple linear regression results for predicting the total PedsQL score based on coping dimensions.

Predictor	β	*p*	95% CI Low	95% CI High
Adaptive coping	19.62	0.081	−2.71	41.96
Avoidant coping	−31.49	<0.05	−60.82	−2.15
Emotional coping	10.98	0.352	−13.31	35.28

**Note.** β = unstandardized regression coefficient; CI = confidence interval. Overall model fit: R^2^ = 0.26, F(3,16) = 1.83, *p* = 0.183.

## Data Availability

The original contributions presented in this study are included in the article. Further inquiries can be directed to the corresponding author.
